# Analysis of Early Assessable Risk Factors for Poor Outcome in Dogs With Cluster Seizures and Status Epilepticus

**DOI:** 10.3389/fvets.2020.575551

**Published:** 2020-10-09

**Authors:** Giulia Cagnotti, Sara Ferrini, Ugo Ala, Claudio Bellino, Cristiano Corona, Elena Dappiano, Giorgia Di Muro, Barbara Iulini, Ida Pepe, Silvia Roncone, Antonio D'Angelo

**Affiliations:** ^1^Department of Veterinary Science, University of Turin, Grugliasco, Italy; ^2^Istituto Zooprofilattico del Piemonte Liguria e Valle d'Aosta, Turin, Italy

**Keywords:** dog, neurology, epilepsy, seizures, risk factors, prognostics

## Abstract

Status epileptics (SE) and cluster seizures (CS) are serious neurological emergencies associated with poor outcome in epileptic patients. Data on risk factors associated with outcome in epileptic patients affected by CS and SE have not been studied extensively to date. In the present retrospective study potential risk factors predictive of negative outcome in a population of dogs affected by CS and SE were analyzed. Ninety-three dogs were included in the study: 21/93 patients (23%) presented with SE and 72/93 (77%) with CS. Based on multivariate statistical analysis, factors statistically associated with a poor outcome were the occurrence of the first epileptic seizure outside the defined idiopathic interval (6 months−6 years), a condition of hyperthermia at presentation and the absence of previous antiepileptic drugs (AEDs) in case of previous history of seizures. The results of the present study implement data on risk factors associated with poor outcome in dogs affected by CS or SE and can aid in the creation of an *ad hoc* scoring system, similar to systems currently applied in human medicine upon hospital admission to benchmark performances and establish protocols for triage and therapeutic management.

## Introduction

Status epilepticus (SE) and cluster seizures (CS) are serious neurological emergencies that require rapid diagnosis and treatment. They are major risk factors for euthanasia and spontaneous death and associated with poor outcome in epileptic patients (1–5). Risk factors associated with outcome in canine patients affected by SE and CS have not been studied extensively and data are limited (2–7). With this study we wanted to investigate and evaluate risk factors predictive of negative outcome in a population of dogs affected by CS or SE, directly assessable upon admission of patients.

## Materials and Methods

### Study Population and Data Collection

For this study we manually reviewed the medical records of dogs admitted to the Veterinary Teaching Hospital, Department of Veterinary Sciences, University of Turin, between July 2015, and February 2019 for CS or SE. The inclusion criterion was SE or CS confirmed by historical description of owners or visual inspection at admission and/or during the hospitalization. All patients underwent at least one neurological examination performed by a board-certified neurologist (ADA) or a neurologist in training (GC) under the supervision of the board-certified neurologist. In cases of multiple hospital stays because of SE or CS during the study period, only the first stay was used for analysis. Data extracted from the charts included: sex, neutering status, breed, size, brachycephalic morphology, age at hospitalization, age at first seizure, seizure type (focal vs. generalized), seizure presentation (CS or SE), emergency treatment protocol, history of previous seizures, previous antiepileptic drug (AED) therapy in epileptic patients, seizure etiology, comorbidity, in-hospital complications, lactate and glucose blood concentration at admission, rectal temperature at admission, heart rate and respiratory rate and pattern at admission, and outcome. Outcome was defined as death in hospital or hospital discharge. Missing medical chart data was a reason for exclusion from the study.

### Definitions

#### Signalment and Age at Hospitalization

Signalment information was collected from the patient's medical records. The patients were classified by sex and reproductive status: intact males, neutered males, intact females, and neutered females. Breed and brachycephalic morphology were defined according to American Kennel Club (https://www.akc.org/) and UK Kennel Club (https://www.thekennelclub.org.uk/) definitions. To determine whether brachycephalic morphology was present in cross breeds, we called the owners and asked whether the dog's muzzle was comparable to that of a typical brachycephalic breed (e.g., pug). Age at hospitalization was expressed in months.

#### Comorbidity

Comorbidity was defined as the presence of disease(s) other than the index disease in an individual (8). Medical conditions observed were classified into cardiac, respiratory, urinary, infectious and parasitic, gastro-enteric, respiratory, gastro-enteric, endocrine, neoplastic, ophthalmologic, neurologic, and locomotor disorders.

Comorbidities were also classified by their impact on the patient's health status: those likely to be incidental and those possibly affecting current health status and/or seizure control. Clinical chart review and classification of diseases was performed by two of the authors (GC and ADA) blinded to patient outcome.

#### Presentation

SE and CS were defined according to the International Veterinary Epilepsy Task Force consensus report. SE was defined as a seizure activity lasting more than 5 min or as the occurrence of two or more epileptic seizures without complete recovery of consciousness in between. CS were clinically defined as the occurrence of two or more epileptic seizures within 24 h (9).

#### Type of Seizure Activity

Seizure type was categorized as focal or generalized. Criteria for categorization as focal seizure included clinical signs (motor, autonomic or behavioral signs, alone or combined) in relation to the function of the area affected by abnormal electrical neuronal activity within one hemisphere. Generalized epileptic seizure was defined as seizure activity affecting both sides of the body and resulting from bilateral involvement of the cerebral hemisphere (9). Only convulsive epileptic seizures (tonic-clonic, tonic, clonic, and myoclonic) were evaluated for this study because electroencephalographic confirmation of non-convulsive seizure activity was unavailable.

#### Complications

In-hospital complications were defined as secondary complications that developed during hospital stay (10).

#### Age at First Seizure, Idiopathic Interval and History of Previous Seizures

History of prior seizures and age at first seizure (expressed in months) were recorded. As seizure onset between age 6 months and 6 years is commonly assumed to be more probably associated with a diagnosis of idiopathic epilepsy (11), this time period was defined as the idiopathic interval.

#### Previous AED Therapy

Information on previous antiepileptic treatment was collected from the medical records of patients with a history of seizures.

#### Seizure Etiology

Data regarding seizure etiology were collected from medical records.

Seizure etiology included: reactive seizures, idiopathic epilepsy (Tier I and Tier II) and structural epilepsy (suspected or confirmed). Reactive seizures were suspected based on a history of possible exposure to toxic agents or based on the results of hematological laboratory tests (9). A diagnosis of idiopathic epilepsy was made according to the International Veterinary Epilepsy Task Force consensus report (Tier I and Tier II confidence level) (11). Structural epilepsy was diagnosed when reactive causes of seizures were excluded, along with signalment, history, an abnormal interictal neurological examination (suspected structural epilepsy) and magnetic resonance imaging and cerebrospinal fluid analysis results (confirmed structural epilepsy) (11).

However, considering the aim of the present study this variable was not taken into consideration for statistical analysis, since seizure etiology is not always available upon admission for all epileptic patients before diagnostic investigations are performed.

#### Lactate and Glucose Concentration

A venous blood sample was taken upon admission during systemic stabilization of patients and analyzed by means of a blood gas analyzer (ABL825 Flex Blood Gas Analyzer, Radiometer Medical ApS, Denmark). Lactate blood concentration reference values were between 2.7 and 22.5 mg/dL (0.3–2.5 mmol/L) (12). Hyperlactemia between 23.4 and 54 mg/dL (2.6 and 6 mmol/L) was defined as mild to moderate; lactate blood concentrations > 54 mg/dL (6 mmol/L) were defined as severe hyperlactemia (12, 13). Glucose blood concentration reference values were between 60 and 130 mg/dL (14). Blood levels below the lower limit indicated hypoglycemia and levels above the upper threshold indicated hyperglycemia.

#### Rectal Temperature, Heart Rate and Respiratory Rate and Pattern

Variables were measured upon admission, during systemic stabilization of patients and classified according to reference values based on size: dogs weighing 4–10 kg were classified as small; the normal range for temperature was 38.5–39.2°C, the heart rate was 80–180 beats per minute (bpm), and the respiratory rate was 24–36 breaths per minute. Dogs weighing 11–25 kg were categorized as medium; the normal range for temperature was 38–39°C, the heart rate was 80–160 bpm, and the respiratory rate was 20–32 breaths per minute. Dogs weighing > 25kg were classified as large; the normal range for temperature was 37.5–38.5°C, the heart rate was 60–80 bpm, and the respiratory rate was 16–25 breaths per minute. In puppies (dogs up to age 9 months), the normal range for temperature was 38.5°C for the first month and then according to size as described for adults, the heart rate up to 220 bpm, and the respiratory rate from 20 to 40 breaths per minute (15). Furthermore, signs of dyspnea were also recorded. Size classification was based on body weight and standard size for breed; reference values were taken from the Royal Canine guidelines (https://www.royalcanin.com/us).

#### Outcome

Poor outcome was defined as death in hospital by euthanasia or spontaneous death. Survivors were discharged after 24 h of being seizure-free or after at least 24 h after discontinuation of drug-induced coma in the absence of seizure activity and according to self-sufficiency.

### Statistical Analysis

Data were analyzed using commercially available software programs [Excel [Microsoft] version 16.27 [19071500]; R commander graphical interface R version 3.3.2 [2016-10-31]]. Standard descriptive statistics are reported as median and interquartile range (IQR) or mean and standard deviation (± SD) for continuous variables and percentage and frequency for categorical variables. Continuous variables were tested for normality distribution using the Shapiro-Wilk test and revealed a non-normal distribution. Numerical data were tested using the Wilcoxon ranked-sum test, while categorical variables were compared using the Chi-square or Fisher's two-tailed exact tests when appropriate. Categorical variables were: sex and neutering status, breed, size, brachycephalic morphology, seizure presentation, comorbidity, complications, idiopathic interval, history of previous seizures, previous AED therapy, seizure type, and respiratory rate and pattern; continuous variables were age at first seizure, age at hospitalization, rectal temperature, heart rate, glucose and lactate blood concentrations. Rectal temperature, heart rate, glycemic level, and blood lactate level were categorized for statistical purposes, as previously reported. Statistical significance was set at *P* < 0.05. A *P*-value between 0.05 and 0.10 was defined as a trend. Variables were also assessed individually with univariate logistic models. Following these analyses, variables with a *P* < 0.10 were entered in multivariate analysis models to identify the most explicative model. Models were compared by analyzing the level of fitness of the model with McFadden's pseudo R squared. Factors were considered significant when the *P* < 0.05.

## Results

A total of 134 dogs were presented for CS or SE during the period of study. Ninety-three patients met the inclusion criteria. Thirty-six (39%) of the final population of 93 dogs were female (19 intact, 17 neutered) and 57 (61%) were male (51 intact, six neutered). The most common breeds were cross breed (32/93), German Shepherd (8/93), and French Bulldog (7/93), followed by three dogs of the breed: American Staffordshire Terrier, Boxer, Chihuahua, Corso dog, two dogs of the breed: Yorkshire, Border Collie, Cocker Spaniel, English Bulldog, Poodle, and Pekinese, and one dog of the breed: Australian Shepherd, Beagle, Bernese Mountain dog, Epagneul Breton, Cavalier King Charles Spaniel, Czechoslovakian Wolf, Dachshund, Dogue de Bordeaux, Pinscher, Giant Schnauzer, Golden Retriever, Labrador Retriever, Maltese, Miniature Schnauzer, Pitbull Terrier, Pomeranian Spitz, Pug, Shih tzu, Siberian Husky, St. Bernard, and Weimaraner. The sample included 27/93 (29%) brachycephalic dogs and was categorized by size in: 34/93 (36%) large, 23/93 (25%) medium, and 36/93 (39%) small breed dogs.

The median age at hospitalization was 88 months (IQR 48–134); the median age at first seizure was 72 months (IQR 24–120). The age at first seizure was within the idiopathic interval in 46/93 dogs (50%). The first seizure occurred before 6 months of age in 4/93 (4%) and after age 6 years in 43/93 (46%). History of previous seizures was recorded in 52/93 dogs (56%), 25 of which were on previous AED treatment for seizure control. In detail, 19 were receiving 1 medication: 14 with phenobarbital (PB), four with imepitoin, and one with gabapentin; four dogs were receiving 2 AEDs: two with PB and levetiracetam, one with PB and carbamazepine, and one with PB and potassium bromide; two were receiving three AEDs: one with PB, imepitoin, and diazepam, and one with PB, potassium bromide, and levetiracetam. In all but one of these 25 cases, the minimal duration of AED therapy before hospitalization was at least 1 month. The duration of previous AED therapy was unknown in 1 patient. The median time between age at first seizure and age at hospitalization was 12 months (IQR 7–22) in the patients on antiepileptic treatment and 9 months (IQR 1–34) in those not receiving an AED.

Forty-five of 93 patients were diagnosed with structural epilepsy (20/45 confirmed and 25/45 suspected), 27/93 with idiopathic epilepsy (8/27 Tier II confidence level and 19/27 Tier I confidence level), and 14/93 with epilepsy of unknown origin. Seven out of 93 patients were diagnosed with reactive seizures (1/7 metaldehyde intoxication, 6/7 hypoglycemia).

A total of 21/93 patients (23%) presented with SE and 72/93 (77%) with CS. Generalized and focal seizures occurred in 87/93 (94%) and 6/93 (6%) dogs, respectively.

The emergency treatment protocol was the same for all patients included in the study: rectal/intravenous diazepam (1–2 mg/kg if the patient was seizuring at presentation) followed by intravenous phenobarbital (4–5 mg/kg q8 h).

Comorbidities were present in 27/93 (29%) dogs; in-hospital complications developed in 6/93 (6%). Complications included: infection (3/6), acute lung injury (1/6), acute kidney insufficiency (1/6), atrial fibrillation and cardiac arrest (1/6).

The median blood glucose level was 109 mg/dL (IQR 96–127); hyperglycemia (median 142.5 mg/dL; IQR 135–162) was recorded in 20/93 (21%) dogs and hypoglycemia (median 49 mg/ dL; IQR 35–53) in 9/93 (10%). The median lactate blood level was 20.7 mg/dL (IQR 12.6–35.1); mild hyperlactatemia (median 35.1 mg/dL; IQR 27.9–41.4) was recorded in 33/93 (35%) dogs, and severe hyperlactatemia (median 94.6 mg/dL; IQR 72–133.3) in 9/93 (10%).

The median temperature was 38.6°C (IQR 38.2–39.2°C). Hyperthermia (median 39.6°C; IQR 39.1–40°C) was recorded in 34/93 (37%−18/34 large size, 10/34 medium size, and 6/34 small size) and hypothermia (median 38°C; IQ: 37.8–38.2°C) in 17/93 (18%−1/17 medium size, 16/17 small size dogs). The median heart frequency was 120 bpm (IQR 100–140); tachycardia (median 120 bpm; IQR 100–140) was recorded in 33/93 dogs (36%−30/33 large size, 3/33 medium size), and bradycardia (median 71 bpm; IQR 67.5–72.5) in 4/93 (4%−3/4 large size and 1/4 medium size). Tachypnea was recorded in 71/93 (76%−30/71 large size, 18/71 medium size, 23/71 small size) and bradypnea in 6/93 (7%−1/6 medium size, 5/6 small size); a dyspneic respiratory pattern was present in 1 dog (1%).

Seventy-two (77%) patients were discharged alive from the hospital, while 21/93 (23%) died during hospital stay: 5 of spontaneous death (cardiorespiratory arrest), accounting for 5% of the study population, and 16 (17%) by euthanasia. None were euthanized for economic reasons but rather because of the persistence of uncontrolled seizure activity despite treatment or severe worsening of the neurological condition. Only one dog that recovered from the seizure activity was euthanized by the owner's request because diagnosed with metastatic lung disease prior to admission.

Statistical analysis revealed a trend for the difference in age at first seizure between survivors (median 61 months, IQR 24–100) and non-survivors (median 120 months, IQR 43–156) (*P* = 0.05). Furthermore, comparison of the outcome in patients with an age at first seizure inside or outside the idiopathic interval showed a mild association: death was more frequent among patients experiencing the first epileptic seizure outside the defined idiopathic interval [*P* = 0.09; odds ratio [OR] 2.34; confidence interval [CI] 0.77–7.72]. Hospital deaths were recorded for 2 of the 4 (50%) that had experienced their first episode before age 6 months, 13 of the 43 (30%) that had experienced their first seizure after 6 years of age, and for 6 of the 46 (13%) for which the age at first seizure was within the idiopathic interval.

The in-hospital complications rate was higher among patients with poor outcome (*P* = 0.02; OR 7.9; CI 1.05–95.15).

Comorbidity stratification revealed an association with outcome: fatal outcome was almost 17 times higher in patients with comorbidity affecting current health status or control of seizure activity than in those with incidental comorbidities (*P* = 0.02).

The median rectal temperature was 38.5°C (IQR 38.2–39°C) and 38.9°C (IQR 38.5–40°C) in survivors and non-survivors, respectively; this difference was statistically significant (*P* = 0.02). When we stratified temperature in low, normal, and high by body size, the risk of death was higher in patients with hyperthermia (*P* = 0.08).

In patients with a history of seizures, those not receiving previous AED therapy were more likely to experience a poor outcome (*P* = 0.01; OR 11.5, CI 1.38–545.73). No other associations were found (Supplementary Table 1).

In order to build a predictive model with the most informative variables, univariate logistic analyses confirmed the associations highlighted by statistical analysis: age at first seizure (*P* = 0.03, OR 1.01, CI 1.00–1.02), previous AED treatment (*P* = 0.02, OR 12.00, CI 1.99–232.56), rectal temperature (*P* = 0.04, OR 3.27, CI 1.11–10.64), presence of comorbidities (*P* = 0.02, OR 16.7, CI 2.02–416.67), and complications (*P* = 0.02, OR 8.23, CI 1.48–63.06) were statistically associated with outcome. A trend was found for age at first seizure inside or outside the idiopathic interval (*P* = 0.09, OR 2.38, CI 0.88–6.90). Based on statistical criteria these variables underwent further analysis. Table 1 presents the associations and their statistical values.

**Table 1:** Univariate analysis.

**Univariate analysis**	**Estimate**	**OR**	***P*-value**	**95% CI (2.5–97.5%)**
Previous AED in epileptic patients (no vs. yes)	2.48	1.25	0.02	1.99–232.56
Age at first seizure (months)	0.10	1.01	0.03	1.001–1.02
Complication (yes vs. no)	2.10	8.23	0.02	1.48–63.06
Temperature (hyperthermia vs. normothermia)	1.19	3.27	0.04	1.11–10.64
Comorbidity (affecting health vs. incidental)	2.89	16.7	0.02	2.02–416.67
Idiopathic epilepsy interval (outside vs. inside)	0.86	2.38	0.09	0.88–6.90

The most explicative and clinically most significant multivariate model was selected as the final one. Rectal temperature, idiopathic interval, and previous AED treatment were the only variables that retained significance: hyperthermia (*P* = 0.005, OR 6.67, CI 1.84–27.67), first seizure outside the idiopathic interval (*P* = 0.04, OR 3.57, CI 1.11–13.33), and absence of previous AED therapy in dogs with a history of seizures (*P* = 0.01, OR 16.67, CI 2.90–436.61) were associated with death (Table 2). Further analyses to evaluate specific associations between variables and clinical presentation revealed a statistically significant association between hypoglycemia and SE presentation (*P* = 0.03, OR: 5.82; CI: 1.07–34.54) and between hyperthermia and SE presentation (*P* = 0.03, OR: 6.48; CI: 1.64–30.66) (Supplementary Table 2).

**Table 2:** Multivariate model.

**Multivariate model**	**Estimate**	**OR**	***P*-value**	**95% CI (2.5–97.5%)**
First seizure outside idiopathic interval	1.28	3.57	0.04	1.11–13.33
Hyperthermia vs. normothermia	1.88	6.67	0.005	1.84–27.67
No previous AED in epileptic patient	3.02	16.67	0.01	2.90–436.61

## Discussion

The early assessable parameters associated with a negative outcome in dogs with CS or SE were absence of a previous AED therapy in dogs with a history of seizures, age at first seizure outside the defined idiopathic interval, and elevated rectal temperature at presentation. To our best knowledge, the literature on risk factors for spontaneous death or euthanasia in epileptic dogs is scant, and information on dogs affected by CS or SE is even more limited (2–7). With the present study we were able to identify several risk factors, directly assessable upon hospital admission, associated with poor prognosis in dogs affected by CS or SE.

Age at seizure onset outside the idiopathic interval was found to predispose epileptic patients to a poor outcome. This may be explained by the underlying etiology of seizure activity. A presumptive diagnosis of idiopathic epilepsy has been associated with the occurrence of the first epileptic seizures between age 6 months and 6 years, along with other diagnostic parameters such as unremarkable interictal clinical and neurological examination and unremarkable blood tests (11). When seizure activity is detected for the first time outside this time interval, we may assume that the patient is more probably affected by structural epilepsy, also in the absence of neurological deficits on interictal neurological examination (16); this etiology has been reported to be associated with a worse outcome (4, 5, 17). Patients with reactive seizures were potentially included in the present study, which may constitute a study bias since reactive seizures can occur independent of age and not necessarily associated with other neurological abnormalities besides seizures (18).

Among the patients with a history of seizures, not receiving AED treatment was more than 16 times more frequent in those with a poor outcome. In human medicine, evidence indicates that early initiation of seizure treatment improves the outcome of patients affected by SE (19–21), and this concept can be reasonably extended to veterinary patients as well (22). Patients who experience recurrent seizures prior to initiation of treatment are, in fact, more likely to develop pharmacoresistant epilepsy due to kindling effects (23). The absence of AED treatment in the patients with a history of seizures might also have been due to the fact that the first episode occurred shortly before hospitalization. But this does not seem to be entirely the case because the mean length of time between first seizure and hospitalization was similar for the patients with a history of seizures and receiving AED therapy and those not receiving antiepileptic treatment. In brief, a plausible reason for not receiving AEDs despite a history of seizures could be lack of responsible management by the owner or a low frequency of seizure episodes.

Polytherapy has been associated with a higher risk of sudden, unexpected death in epilepsy in human medicine, though it has also been postulated that this association may be a marker for severity of epilepsy (24). We found no statistically significant difference in outcome of epileptic dogs receiving one or more AEDs; this finding is shared by previous studies (5).

Among the clinical vital signs recorded in an emergency setting, the only statistically significant variable we identified was rectal temperature. Increased rectal temperature was more frequently recorded in patients who died during hospital stay. Hyperthermia can result from severe muscular contraction secondary to seizure activity: the longer the duration of seizure activity, the higher the body temperature rises, and the worse the clinical condition. Also, prolonged muscle contractions and hyperthermia can lead to rhabdomyolysis and myoglobinuria, which may compromise renal function when co-occurring with hypotension and severe metabolic acidosis (22, 25, 26). Similar conclusions can be drawn for the association found between hyperthermia and SE presentation, even if rhabdomyolysis and clinical consequences were not detected in any of the cases in the study.

Univariate statistical analysis showed an almost eight-fold higher likelihood of short-term poor outcome among the patients who developed in-hospital complications; however, this variable did not retain significance on multivariate statistical analysis. The difference can be explained by the low number of patients that developed in-hospital complications. Since infection, cardiac dysfunction, and respiratory failure are complications of prognostic importance in SE outcome in humans (27, 28), it is reasonable to assume that complications can worsen the outcome of a severe neurological condition by worsening a patient's clinical status.

We evaluated comorbidities because they are a variable of interest in a scoring system developed in human medicine for patients with SE (29). Methods for measuring comorbidity in human medicine are classified as either a “disease count” (enumeration of the number of conditions present) or an “index.” Among the human indexes that have been developed, some rate the comorbidity burden according to a system that assesses the effect of the condition on specific body systems, while others list clearly defined diagnoses. In both methods the conditions are further described by ranking the severity (30–32). Although the data in human literature show that indexes are more accurate than a simple count, such indexes have not been defined for veterinary patients. As the comorbidities recorded in our study sample differed from those included in human index lists, a simple count was made. Analysis showed no association between comorbidity and outcome when we compared the dogs with comorbidities (one or more) and those without comorbidities or when we made a disease count. We also categorized the comorbidities into those likely to be incidental and those possibly affecting current health status and/or seizure control. The latter category was more frequently detected in patients displaying a poor outcome. As for the development of in-hospital complications, comorbidities affecting current health can worsen the outcome of a severe neurological condition by worsening a patient's clinical status.

Despite the clear evidence that SE is a more severe condition than CS (22, 26, 33), we found no significant association between clinical presentation and outcome. Both conditions can vary in severity, duration (SE and CS), and frequency (CS). Statistical analysis was not performed to investigate this aspect, however, because the information on seizure duration and frequency was often inconsistent in the clinical chart notes based on the owners' reports, and the retrospective nature of the study precluded the possibility to obtain reliable data regarding these variables.

Older age at hospitalization was not found to be associated with outcome. A bias that might have influenced this result is the fact that smaller breeds generally live longer than larger breeds, so age burden may differ among breeds and sizes (34). In the present study sample, there were no significant differences in the distribution of small and large breed dogs for the total sample or between survivors and non-survivors, so it is unlikely that this confounding factor influenced our results.

A history of prior seizures was evaluated as a potential prognostic factor: the Status Epilepticus Severity Score is a prognostic score for human patients with SE. It relies on four significant outcome predictors, including previous history of seizures (35, 36). In this scoring system, the absence of previous seizures is considered a surrogate of acute symptomatic etiology and a risk factor for death (36). We found no similar association, however. The majority of the dogs in our study had a history of generalized seizures; we found no association between type of seizure activity and outcome. We did not divide generalized seizures into their various subtypes (tonic-clonic, clonic, tonic, atonic, or absence) nor did we distinguish focal seizures in motor, behavioral, and autonomic. Finally, we did not assess the specific weight of clinical manifestations of seizure activity on outcome. SE can be further classified as either convulsive or non-convulsive. Although non-convulsive generalized SE is associated with a worse outcome according to human scoring systems (36), such seizure activity can be diagnosed only by means of electroencephalographic monitoring, a tool not available in many veterinary services and not routinely performed at our institution. For these reasons, this variable was not included in our analysis.

Neither glucose nor lactate blood level was statistically associated with outcome. The association between elevated lactate plasma concentration at admission and mortality has been well-documented in dogs, with non-survivors noted to have significantly higher lactate concentration compared to survivors. Furthermore, lactate concentration is a significant predictor of mortality in the acute patient physiologic and laboratory evaluation scoring system, a veterinary disease severity scoring system (37, 38). It has been reported, however, that subsequent lactate level measurement outperformed the single measurement at admission, so it is now clear that a greater risk of death is associated with persistent hyperlactatemia rather than a single time point finding of elevated lactate concentration (38). These observations may explain the absence of a significant result in our study, given that lactate concentrations were evaluated only at admission and further measurements were not entered into the analysis.

Hyperglycemia is another predictor variable in the acute patient physiologic and laboratory evaluation scoring system, though the authors concluded that stress hyperglycemia was more probably the manifestation of an epiphenomenon rather than a truly causative factor for mortality (37). In neurology, and in head trauma patients in particular, hyperglycemia has been associated in both human and veterinary medicine with injury severity but not outcome (39–41). Our study results seem to confirm these findings.

While we found no statistically significant association between sex and outcome, there was a slightly higher prevalence of epileptic seizures among the male dogs (42, 43). Several studies have reported conflicting data on the predisposition to seizures of spayed compared to sexually intact females (4, 7); we found no association that demonstrated a significantly higher risk of developing seizures in intact compared to neutered females.

The mortality and survival rates of dogs with CS and SE in the present study are consistent with previous studies that applied similar inclusion criteria (7). Some reported a higher in-hospital mortality of 38.5% albeit based on a higher rate of euthanasia (33% among hospitalized dogs) (4). The percentages of short-term mortality due to spontaneous death are comparable: 5.4 and 5.1% in our study and in the study by Zimmermann and colleagues, respectively. Bateman et al. described similar percentages as well: 2.1% died spontaneously and 23.2% were euthanized (4, 7).

In our study, hypoglycemia at presentation was associated with SE presentation. The role of glucose in the pathophysiology of epileptic seizures, and in SE in particular, is debated. Energy deprivation via either hypoxia or hypoglycemia often results in coma and neuronal death and it is sometimes associated with the onset of seizure activity (44, 45). Nevertheless, the human literature reports that several systemic changes can occur during SE, including a decrease in glucose blood concentration (20). From this point of view, hypoglycemia may not be a cause but rather a consequence. Furthermore, *in vitro* recordings have shown that low glucose levels might also affect seizure duration, reducing the frequency and the amplitude in the seizure-like discharge by 50 and 25%, respectively (46). According to the ketogenic diet rationale, increasing calorie restriction results in improved seizure control in epileptic mice; though the exact mechanisms underlying its clinical efficacy remain unknown (47). In veterinary medicine, dogs suffering from reactive seizures (frequently caused by hypoglycemia) were reported to have a 1.57 higher odds for developing SE than dogs with idiopathic epilepsy (6).

The main limitation of our study is that both euthanasia and spontaneous death were defined as poor outcome. The degree to which the frequency of euthanasia outweighs “natural” death in veterinary medicine poses a unique challenge to models developed on mortality outcome, as it is mainly influenced by owner disposition (48). We attempted to overcome this limitation by examining the reasons for euthanasia. The owners' attitude and economic status did not influence the outcome in any case. They opted for euthanasia due to the persistence of uncontrolled seizure activity despite treatment or to severe worsening of the neurological condition. Only one patient was euthanized because of concurrent severe systemic disease. In addition, the owner's personal attitude, financial situation, and lifestyle may have influenced the greater or lesser effectiveness of AED therapy and the report of a previous history of seizures.

Inclusion of seizure etiology among the variables would have certainly enhanced the value of our study, but very often seizure causes can only be detected after ancillary diagnostic exams, requiring a delay of up to several hours or days after admission, which may be critical for therapeutic management. The aim of this study was to evaluate the clinical and demographic features that can be assessed at hospital admission.

With this study we wanted to identify risk factors associated with short-term outcome in dogs with CS or SE. Multivariate analysis showed that age at first seizure outside the idiopathic interval, absence of previous AED therapy in dogs with a history of seizures, and increased rectal temperature were the only factors that were statistically different between the dogs that survived and those that did not. Recently developed diagnosis-independent veterinary scores can be used to benchmark performance and establish protocols for triage and therapeutic management. Appropriate reference scores can be used for objective measurement of illness severity and improve analysis of treatment outcome significantly (48). With this in mind, the present study was designed to implement data for the creation of an *ad hoc* scoring system, similar to systems currently applied in human medicine, for use upon hospital admission of patients with CS or SE.

## Data Availability Statement

All datasets presented in this study are included in the article/Supplementary Material.

## Ethics Statement

Ethical review and approval was not required for the animal study because of the retrospective nature of the study. Written informed consent was obtained from the owners for the participation of their animals in this study.

## Author Contributions

GC and AD'A: study design, data collection, statistical analysis, interpretation of results and generation of the manuscript, critical revision, and final approval of version to be published. SF: data collection, statistical analysis, interpretation of results and generation of the manuscript, critical revision, and final approval of version to be published. UA and CB: statistical analysis, interpretation of results, critical revision, and final approval of version to be published. CC, ED, GD, BI, IP, and SR: data collection, critical revision, and final approval of version to be published. All authors contributed to the article and approved the submitted version.

## Conflict of Interest

The authors declare that the research was conducted in the absence of any commercial or financial relationships that could be construed as a potential conflict of interest.

## Abbreviations

SE: Status Epilepticus
CS: Cluster Seizures
AED: Antiepileptic Drug
IQR: Interquartile Range
SD: Standard Deviation
PB: phenobarbital
CI: Confidence Interval
OR: Odd Ratio.
