# Supplementary material for: Analysis of Early Assessable Risk Factors for Poor Outcome in Dogs With Cluster Seizures and Status Epilepticus
Source: Front Vet Sci. 2020 Oct 9;7:575551. doi: 10.3389/fvets.2020.575551 (PMC7581674; doi:10.3389/fvets.2020.575551)
Supplement: Supplementary file 1 [file Table_1.pdf]

Supplementary Material

1 Supplementary Tables

Supplementary Table 1. Cohort characteristics compared to outcome.

**Characteristic – no. (%)**	**Patients N=93 (100)**	**Survivors N=72 (77)**	**Non-survivors N=21 (21)**	**P-value**
**Sex**				0.85
Male	51 (55)	41 (57)	10 (48)	
Male neutered	6 (7)	4 (6)	2 (9)	
Female	19 (20)	14 (19)	5 (24)	
Female neutered	17 (18)	13 (18)	4 (19)	
**Breed**				1.00
Crossbred	32 (34)	25 (35)	7 (33)	
Purebred	61 (66)	47 (65)	14 (67)	
**Size**				0.21
Small	36 (39)	28 (39)	8 (38)	

Medium	23 (25)	15 (21)	8 (38)	
Large	34 (36)	29 (40)	5 (24)	
**Brachycephalic morphology**	27 (29)	23 (32)	4 (19)	0.25
**History of seizures**	52 (56)	42 (58)	10 (48)	0.38
Previous AEDs	25 (48)	24 (57)	1 (10)	**0.01**
AED Count				0.24
Monotherapy	19 (76)	19 (79)	0 (0)	
Polytherapy	6 (24)	5 (21)	1 (100)	
**Presentation**				0.18
Status epilepticus	21 (23)	14 (19)	7 (33)	
Cluster seizures	72 (77)	58 (81)	14 (67)	
**Seizure type**				0.72
Generalized	87 (94)	67 (93)	20 (95)	
Focal	6 (6)	5 (7)	1 (5)	
**Comorbidities**	27 (29)	19 (26)	8 (38)	0.30

Type				**0.02**
No comorbidities	66 (71)	53 (74)	13 (62)	
Incidental comorbidities	22 (24)	18 (25)	4 (19)	
Affecting health status	5 (5)	1 (1)	4 (19)	
Disease count				0.94
One comorbidity	20 (74)	14 (74)	6 (75)	
Two comorbidities	7 (26)	5 (26)	2 (25)	
Disease type				
Cardiac	8	5	3	
Respiratory	4	2	2	
Urinary	1	1	0	
Infectious and parasitic	2	1	1	
Gastro-enteric	3	2	1	
Endocrine	3	2	1	
Neoplastic	3	1	2	
Ophtalmologic	6	6	0	

Neurologic	2	2	0	
Locomotor	2	2	0	
**Complications**	6 (6)	2 (3)	4 (19)	**0.02**
Infection	3	2	1	
Acute lung injury	1	0	1	
Acute kidney insufficiency	1	0	1	
Atrial fibrillation and cardiac arrest	1	0	1	
**Age at first seizure (months, range)**	72 (24 – 120)	61 (24 – 100)	120 (43 – 156)	**0.05**
**First seizure in idiopathic interval**	46 (49)	40 (56)	6 (29)	**0.09**
**Age at hospitalization (months, range)**	88 (48 – 134)	84 (47 – 127)	129 (48 – 156)	0.13
**Serum glucose concentration**				0.65
Normoglycemia	64 (69)	51 (71)	13 (62)	
Hypoglycemia	9 (10)	6 (8)	3 (14)	
Hyperglycemia	20 (21)	15 (21)	5 (24%	
**Serum lactate concentration**				0.61

Normolactemia	51 (55)	38 (53)	13 (62)	
Mild-moderate hyperlactemia	33 (35)	26 (36)	7 (33)	
Severe hyperlactemia	9 (10)	8 (11)	1 (5)	
**Rectal temperature**				**0.08**
Normothermia	42 (45)	36 (50)	6 (29)	
Hypothermia	17 (18)	14 (19)	3 (14)	
Hyperthermia	34 (37)	22 (31)	12 (57)	
**Heart rate**				0.75
Within normal range	56 (60)	42 (58)	14 (67)	
Bradycardia	4 (4)	3 (4)	1 (5)	
Tachycardia	33 (36)	27 (38)	6 (28)	
**Respiratory rate and pattern**				0.31
Eupnea	15 (16)	12 (17)	3 (14)	
Dyspnea	1 (1)	0 (0)	1 (5)	
Bradypnea	6 (7)	5 (7)	1 (5)	
Tachypnea	71 (76)	55 (76)	16 (76)	

Supplementary Table 2. Comparison of patient characteristics and presentation.

Characteristic – no. (%)	**Patient** **N=93 (100)**	**Cluster seizures N=72 (77)**	**Status Epilepticus** **N=21 (23)**	**P-value**
**Sex**				0.34
Male	51 (55)	41 (57)	10 (48)	
Male neutered	6 (7)	3 (4)	3 (14)	
Female	19 (20)	14 (19)	5 (24)	
Female neutered	17 (18)	14 (20)	3 (14)	
**Breed**				0.07
Crossbred	32 (34)	21 (29)	11 (52)	
Purebred	61 (66)	51 (71)	10 (48)	
**Size**				0.28
Small	36 (39)	30 (42)	6 (29)	
Medium	23 (25)	15 (21)	8 (38)	
Large	34 (36)	27 (37)	7 (33)	
**Brachycephalic morphology**	27 (29)	24 (33)	3 (14)	0.11

**History of seizures**	52 (56)	42 (58)	10 (48)	0.46
Previous AEDs	25 (48)	20 (48)	5 (50)	0.70
AED count				0.7
Monotherapy	19 (76)	15 (75)	4 (80)	
Polytherapy	6 (24)	5 (25)	1 (20)	
**Seizure type**				0.61
Generalized	87 (94)	68 (94)	19 (90)	
Focal	6 (6)	4 (6)	2 (10)	
**Comorbidities**	27 (29)	21 (29)	6 (29)	1.00
Disease count				1.00
One comorbidity	20 (74)	17 (81)	3 (50)	
Two comorbidities	7 (26)	4 (19)	3 (50)	
Disease				
Cardiac	8	7	1	
Respiratory	4	3	1	
Urinary	1	0	1	

Infectious and parasitic diseases	2	1	1	
Gastroenteric	3	2	1	
Endocrine	3	3	0	
Neoplastic	3	1	2	
Ophtalmologic	6	5	1	
Neurological	2	2	0	
Locomotor	2	1	1	
Type				0.57
No comorbidity	66 (71)	51 (71)	15 (71)	
Incidental	22 (24)	18 (25)	4 (19)	
Affecting health comorbidities	5 (5)	3 (4)	2 (10)	
**Complications**	6 (6)	5 (7)	1 (5)	1
Infections	3	3	0	
Acute lung injury	1	1	0	
Acute kidney insufficiency	1	0	1	
Atrial fibrillation and cardiac arrest	1	1	0	

**Age at first seizure (months, range)**	72 (24 – 120)	75 (36-125)	49 (12-106)	0.27
**First seizure in idiopathic interval**	46 (49)	33 (46)	13 (62)	0.22
**Age at hospitalization (months, range)**	88 (48 – 134)	89.5 (48-139)	72 (20-125)	0.45
**Serum glucose concentration**				**0.03**
Normoglycemia	64 (69)	53 (74)	11 (52)	
Hypoglycemia	9 (10)	4 (5)	5 (24)	
Hyperglycemia	20 (21)	15 (21)	5 (24)	
**Serum lactate concentration**				0.20
Normolactemia	51 (55)	42 (58)	9 (43)	
Mild-moderate hyperlactemia	33 (35)	22 (31)	11 (52)	
Severe hyperlactemia	9 (10)	8 (11)	1 (5)	
**Rectal temperature**				**0.03**
Normothermia	42 (45)	38 (53)	4 (19)	
Hypothermia	17 (18)	14 (19%)	3 (14)	
Hyperthermia	34 (37)	20 (28)	14 (67)	
**Heart rate**				0.41

Within normal range	56 (61)	44 (61)	12 (57)	
Bradycardia	4 (4)	2 (3)	2 (10)	
Tachycardia	33 (35)	26 (36)	7 (33)	
**Respiratory pattern**				0.13
Eupnea	15 (16)	14 (19)	1 (5)	
Dispnea	1 (1)	0	1 (5)	
Bradypnea	6 (7)	5 (7)	1 (5)	
Tachypnea	71 (76)	53 (74)	18 (85)	
